# New Bases for Artificial Teeth

**Published:** 1870-03

**Authors:** 


### NEW BASES FOR ARTIFICIAL TEETH.

This subject is eliciting the earnest attention of a large class of the Dentists in the country.

Perhaps three-fourths of those who have entered the profession within the last ten years are especially interested in this matter, and the attention and efforts of a large proportion of these are directed to supplying artificial teeth.

Formerly, gold and silver supplied the demand in this direction. Some fifteen or eighteen years ago, platinum came in as the foundation of continuous gum work. Following this a few years, came the rubber for dental plates. For several reasons, which are generally well understood, it was almost universally used, though many have always had serious objections to it. During the last two or three years, such conditions and restrictions have been attached to it as to render it very unpopular with the large proportion of the profession; and they have been for the last year and a half on the lookout for something to supplant the rubber, and if the profession will but avail themselves of either of three or four things that are presented, the use of rubber for artificial dentures may be totally abandoned at once. And in the first place here we will say, that we regard the ordinary silver-plate work equally as good as rubber in every respect, and in some far better; but one difficulty of this style of work is, that not more than one in ten of those now attempting to practice can make practical gold or silver dentures. But, other things are introduced that can be used with greater facility than these—such, for example, as wrought aluminum plates, with the appropriate solders or alloys for attachment; and, in addition to this, there is the cast plates of aluminum, Moffitt's, and Weston's metals, any of which are far preferable to rubber for dental plates.

By either of these materials can plates be as perfectly adapted as by rubber, and sufficient strength secured without the thickness and clumsiness of that material. These materials and processes may be used by all who will purchase the material without license. We have some experience with Weston's metal, at least so far as seeing it after it has been worn in the mouth is concerned, and are very much pleased with it.

Our attention has been called, within a very recent period, to a new material for wrought plates, called platinated silver, prepared by Drs. Watt and Williams. Plates from this metal are gotten up with metal dies, in the usual manner. The attachment for the teeth, together with the rimming, is made by an alloy, having as nearly as possible the same affinities as the platinated silver. This metal, in its fusibility and facility of being cast, is similar to Weston's or Moffitt's metal, and is used by casting.

There are three or four methods of making cheap artificial dentures now open to the profession, any of them as easily manipulated as rubber, and in all respects as good, and in three respects at least, far less objectionable than rubber. We suggest to every member of our profession, who is in the line of cheap Dentistry, try them. T.

